# The evolution of corneal and refractive surgery with the femtosecond laser

**DOI:** 10.1186/s40662-015-0022-6

**Published:** 2015-07-14

**Authors:** Antonis Aristeidou, Elise V. Taniguchi, Michael Tsatsos, Rodrigo Muller, Colm McAlinden, Roberto Pineda, Eleftherios I. Paschalis

**Affiliations:** Laser Institute Thessaloniki, Thessaloniki, Greece; Massachusetts Eye and Ear Infirmary, Department of Ophthalmology, Harvard Medical School, Boston, MA USA; Massachusetts Eye and Ear Infirmary/Schepens Eye Research Institute, Boston Keratoprosthesis Laboratory, Harvard Medical School, Boston, 02114 MA USA; Southampton University Hospitals, Southampton, UK; Flinders University, Adelaide, South Australia Australia; Wenzhou Medical University, Wenzhou, Zhejiang China

**Keywords:** Femtosecond, Laser, Refractive, Corneal surgery, Cataract surgery, Penetrating keratoplasty, Deep anterior lamellar keratoplasty, Descemet's stripping automated endothelial keratoplasty, Laser in situ keratomileusis, Small incision lenticule extraction

## Abstract

The use of femtosecond lasers has created an evolution in modern corneal and refractive surgery. With accuracy, safety, and repeatability, eye surgeons can utilize the femtosecond laser in almost all anterior refractive procedures; laser in situ keratomileusis (LASIK), small incision lenticule extraction (SMILE), penetrating keratoplasty (PKP), insertion of intracorneal ring segments, anterior and posterior lamellar keratoplasty (Deep anterior lamellar keratoplasty (DALK) and Descemet's stripping endothelial keratoplasty (DSEK)), insertion of corneal inlays and cataract surgery. As the technology matures, it will push surgical limits and open new avenues for ophthalmic intervention in areas not yet explored. As we witness the transition from femto-LASIK to femto-cataract surgery it becomes obvious that this innovation is here to stay. This article presents some of the most relevant advances of femtosecond lasers to modern corneal and refractive surgery.

## Introduction

Femtosecond laser technology was first developed by Dr. Kurtz at the University of Michigan in the early 1990s [1] and was rapidly adopted in the surgical field of ophthalmology. Femtosecond lasers emit light pulses of short duration (10^−15^ s) at 1053 nm wavelength that cause photodisruption of the tissue with minimum collateral damage [1–3]. This enables bladeless incisions to be performed within the tissue at various patterns and depth with high precision. This review paper presents the most recent advancements in femtosecond lasers in modern corneal and refractive surgery.

## Review

### Femtosecond Laser-Assisted LASIK (FS-LASIK)

Globally, laser in situ keratomileusis (LASIK) is the treatment of choice for the surgical correction of refractive errors, particularly myopia [4]. Although a short rehabilitation period and rapid stabilization of visual outcome are main advantages over photo-refractive keratectomy (PRK), increased incidence of dry eye and flap related complications could have a substantial impact on the patient’s quality of life [5].

#### Advantages in flap creation

The femtosecond laser has revolutionized corneal and refractive surgery with respect to its increased safety, precision, and predictability over traditional microkeratomes. Advantages of bladeless femtosecond assisted LASIK (FS-LASIK) over conventional microkeratome assisted LASIK (MK-LASIK) include reduced dry eye symptomatology, reduced risk of flap button hole or free cap formation [6, 7], and gentler approach with minimal or no transient visual loss (black out period) due to close physiologic maintenance of intraocular pressure (IOP) throughout the procedure, especially in femtosecond laser platforms that employ a curved contact surface. This configuration allows better approximation of the corneal surface and reduced suction pressure compared to flat contact surface laser interfaces and traditional microkeratomes.

#### Flap accuracy and predictability

Kezirian and Stonecipher have reported fewer complications, better flap thickness predictability, and less surgically induced astigmatism in eyes treated with FS-LASIK compared to the Hansatome (Bausch & Lomb, Rochester, New York) and Carriazo-Barraquer (CB) microkeratome (Moria, Antony, France) [8]. Pajic et al. found the femtosecond laser (Technolas) to be superior to the mechanical microkeratome (Amadeus II) in terms of flap thickness predictability and the speed of visual acuity recovery in a prospective, randomized, paired eye study [9]. Flap diameter, thickness accuracy, [9–15] and flap thickness reproducibility [13] have been consistently shown to be superior in femtosecond created flaps compared to microkeratome assisted flap creation. Recently, Zhang et al. [16] investigated the thickness and the morphology of the WaveLight FS200 (Alcon Laboratories Inc., Fort Worth, Texas, USA) femtosecond laser microkeratome compared with microkeratome flaps, using anterior segment optical coherence tomography (AS-OCT). Femtosecond created flaps were found to deliver more accurate, reproducible flaps with uniform thickness compared to those created by the Moria microkeratome (Moria SA, Antony, France). Electron microscopy [17] has shown that the quality of the stromal bed surface in flaps created with the Moria or the 15 kHz IntraLase were comparable in quality and surface characteristics. In addition, the 30 kHz IntraLase provide better stromal bed characteristics compared to Moria and the 15 kHz IntraLase [17].

#### Flap morphology / Advantages in stability, epithelial ingrowth, and stromal surface

The accuracy and reproducibility of femtosecond lasers allow for a thin (100–110 μm) or even thinner uniform (planar) flap compared to the meniscus-shaped flap (thinner centrally and thicker peripherally) created with the manual microkeratome [18]. FS-LASIK flaps have shown greater precision in flap diameter and thickness and a more uniform flap thickness across the flap diameter [19, 20]. Additionally, it allows the surgeon to plan the angulation of the flap periphery, which may provide better flap stability and reduce clinically significant epithelial ingrowth [16, 21, 22]. Another significant advantage of femtosecond lasers is the ability to resume a lamellar flap after loss of suction or technical interruptions, and even when performing a secondary flap underneath a primary of substandard quality with little risk of serious repercussions.

#### Biomechanical stability

The ability to cut thinner flaps with minimal effects on stromal architecture in addition to the option of creating oval flaps with a shorter vertical than horizontal diameter allows cutting of fewer vertical than horizontal lamellae thus reducing the weakening effect of flap creation [18]. Further, the use of a femtosecond laser enables an acute side cut angle, such as 150 ° resulting in less biomechanical insult to the cornea [18]. This is in contrast to microkeratomes that are prone to variation in corneal biomechanics thus creating less uniform flaps, especially for intended thin flaps [23].

Biomechanical studies demonstrate that corneas are more stable with thin uniform flaps compared to thicker flaps [18, 24]. Femtosecond lasers have undergone a number of improvements since their introduction including smaller and more tightly packed cavitation bubbles to enable almost resistance-free stromal bridges, thinner and more predictable flaps with smoother interfaces, and algorithms for the creation of elliptical flaps or flaps with everted edges to enable mechanical stability [25–29]. There are also greater options in flap diameter, flap thickness, side cut angle, hinge position, and hinge length.

### Early and long-term outcomes

#### Visual quality

Tran et al. conducted a prospective, randomized, contralateral study to evaluate aberrations induced following LASIK flap creation only (no excimer ablation) with the femtosecond laser and the Hansatome microkeratome [30]. No increase in high-order aberrations was observed in eyes with femtosecond created flaps whereas a significant increase was noted in the microkeratome group [30]. Moreover, eyes with femtosecond created flaps have shown better contrast sensitivity at high spatial frequencies under both photopic and scopic conditions compared to the microkeratome group (Carriazo Barraquer, Moria, Antony, France) [31].

#### Stability of myopic treatments

A prospective, randomized, contralateral eye study comparing the femtosecond laser and the Hansatome microkeratome found significantly better uncorrected visual acuity outcomes in the early postoperative period (up to 3 months) and less residual postoperative astigmatism in femtosecond eyes [32]. Kanellopoulos and Assimelis looked at 109 consecutive patients that underwent myopic LASIK using the FS200 femtosecond and EX500 excimer laser at 1, 3, 6 and 12 months. They found that 94.7 % of eyes had postoperative unaided visual acuity better than 1.0 (decimal) at month 3, and maintained this till month 12 [33]. Similarly, Han et al. [34] showed remarkable refractive outcomes and stability in myopic LASIK where 98 % of the eyes had a manifest refraction within ±1.0 D at 1 year.

#### Stability of hyperopic treatments

Gil-Cazorla et al. [35] performed a retrospective, nonrandomized, interventional, comparative case series that looked at 72 eyes that had hyperopic LASIK using the 60 kHz IntraLase femtosecond laser and 72 eyes that underwent hyperopic LASIK using the Moria M2 microkeratome. They found that the FS-LASIK group had statistically significantly lower mean residual sphere and better uncorrected visual acuity compared to the microkeratome group. Another study looked at topography-guided hyperopic FS-LASIK using the IntraLase FS60 and Wavelight FS200 with the Wavelight 400Hz excimer laser in 202 eyes over a follow up period of 24 months. Although this was not a comparative study, it showed remarkable stability and effectiveness with no significant changes in aberrations [36].

#### Epithelial remodeling

Epithelial remodeling after keratorefractive treatment has been recognized for a considerable time [37, 38]. Although there is no study to date that directly compares epithelial remodeling following FS-LASIK with conventional MK-LASIK, the plausible explanations for epithelial remodeling, such as the rate of stromal curvature change [39] and the change in biomechanical stability especially in large corrections [40] should have a much smaller impact compared to MK-LASIK.

#### Dry eye

Dry eye remains a common and important post-LASIK complication with up to 90 % of LASIK patients experiencing dry eye symptoms [41–43]. Salomão et al. looked at dry eye symptoms, signs, and severity with the IntraLase femtosecond and Hansatome LASIK. They found that significantly less patients suffered dry eye symptoms and had less evidence of superficial punctate epithelial erosions in the IntraLase group 1-month post LASIK [44]. There were also significantly less patients needing topical cyclosporine in the IntraLase group. The authors postulated that patients in the IntraLase group incurred less goblet cell damage than in the Hansatome group. This could be attributed to the higher IOP required for microkeratome (Hansatome) flap creation. Additionally they speculated that the thinner flaps created with the IntraLase were responsible for less afferent nerve damage in the anterior corneal stroma that may influence dry eye symptomatology. In contrast to this study, Golas et al. [45] performed a randomized clinical trial that included 51 patients that had wavefront-guided LASIK using a mechanical microkeratome in 1 eye and a femtosecond laser in the fellow eye. There was no statistically significant difference in self-reported dry eye symptoms at 1, 3, 6 and 12 months post LASIK between the two groups.

### Femtosecond laser-specific complications

The incidence of complications such as button-hole, epithelial abrasion, incomplete flap, free cap, Bowman stripe, and irregular cuts are substantially reduced with femtosecond lasers. However, there are some complications specific to femtosecond lasers such as cavitation gas bubbles (known as opaque bubble layer (OBL)) that tend to disappear within minutes. Modifications in flap design can reduce their incidence [46] but their presence can impede the surgeon and the excimer laser’s eye tracker to visualize and locate the pupil respectively. Extreme OBL can result in intracameral bubbles as well. The exact origin of these bubbles is unknown. The most credible theory suggests that they originate from stray laser pulses into the aqueous humor [47]. Another thought is migration of the corneal stromal gas bubbles retrograde through Schlemm’s canal into the anterior chamber. The bubbles can be moved away from the visual center with gentle cannula manipulations. Alterations in pulse duration that enable reduction in collateral tissue damage could help to reduce the formation of cavitation bubbles [48].

Transient light sensitivity syndrome (TLSS) is also another femtosecond laser specific complication usually encountered within the first few weeks of the femtosecond LASIK procedure. It is characterized by photophobia of variable severity associated with little or no corneal inflammation [49]. It is believed to be due to a biochemical response of corneal keratocytes to near-infrared laser energy or an inflammatory response of the adjacent tissues to gas bubbles [50]. Although no inflammation is evident, intensive topical steroid in the immediate post-operative period appears to reduce the incidence of TLSS from 2.8 % to 0.4 % in a study by Munoz et al. [49]. Another interesting finding in this study was that diffuse lamellar keratitis (DLK) was more likely to occur in eyes with TLSS (30 %) compared to eyes without TLSS (3 %). The incidence of TLSS appears to decrease as femtosecond laser frequency increases allowing the use of less energy for flap creation [50].

Rainbow glare is another femtosecond LASIK-related complication, induced from light scattering at the posterior surface of the interface. Krueger et al. first described it in 2008 [51]. Patients described seeing between 4 and 12 bands of color and this phenomenon has no predilection to age, gender, or refractive error. However, Bamba et al. [52] found a positive correlation between rainbow glare and increased laser energy used. The incidence of rainbow glare appears to have faded with the newer generation of femtosecond lasers that provide improved focusing optics [51–53].

DLK also known as “Sands of the Sahara” syndrome, diffuse interface keratitis, or diffuse interstitial keratitis, is a sterile inflammatory reaction that typically occurs one week after LASIK [54]. Gil-Cazorla et al., Chan et al. and Morshirfar et al. [7, 55, 56] found a higher incidence of DLK in femtosecond (IntraLase) performed LASIK compared to Moria and Hansatome microkeratomes respectively. This increased rate of DLK seems to be attributed to higher flap interface inflammatory response due to laser energy and gas bubbles that cause increased activation of anterior stromal keratocytes as seen by confocal microscopy [11].

### Femtosecond refractive and small incision lenticule extraction

**Fig. 1:**
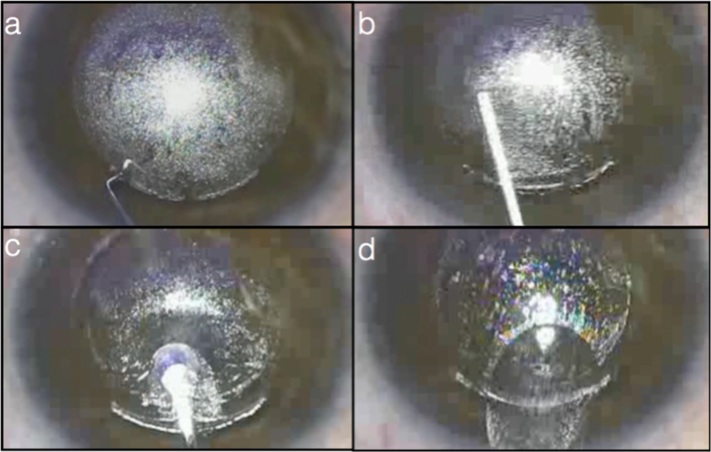
RELEX-SMILE procedure. **a.** The flap has been created and a spatula is used to dilate the incision. **b.** The lenticule is detached first from the anterior and then from the posterior stroma. **c.** The lenticule is being caught and (**d**) removed through the small incision. The removal of the lenticule results to a change in corneal thickness, which in turn results to a refractive power change of the cornea

In its current configuration, FS-LASIK still requires two laser platforms – one for flap creation (femtosecond laser) and another for stromal bed ablation (excimer laser), which naturally affects the time required in the laser suite and cost of the laser procedure. In 2008, Refractive Lenticule extraction (ReLex) was introduced in order to utilize one femtosecond laser platform for flap creation and refractive lenticule extraction (Fig. 1). This would reduce treatment time as the complete procedure is performed with one laser platform, avoiding transfer of the patient from one laser platform to another. Soon after that, in 2009, both ReLex and its variation, small incision lenticule extraction (SMILE), obtained CE approval [57–59]. SMILE potentially offers more advantages than ReLex as it does not require the creation of a flap thus avoiding flap-related early or late complications and yet improving patient experience over conventional microkeratome assisted LASIK with no transfer between laser platforms. The absence of flap creation with minimal disruption of the anterior stromal architecture as the corneal lenticule is extracted from the mid stroma allows for much greater preservation of biomechanical integrity and stability of the cornea [16, 24]. The minimal disruption of the anterior corneal surface epithelium, Bowman’s layer and anterior stroma may be associated with less risk of dry eye. In the human cornea, nerve fibers run from the periphery in the anterior third of the stroma towards the center in a radial fashion [60, 61] and then penetrate Bowman's layer and branch vertically and horizontally between the Bowman’s and basal epithelium to create a network of nerve fibers known as the sub-basal nerve plexus. This plexus is particularly damaged in LASIK with the creation of the flap and further affected with the excimer laser ablation. Compared to SMILE, the basal nerve plexus is minimally disrupted with significantly less risk of dry eye and patient discomfort [61–65].

A number of prospective studies have shown equivalence if not superiority of RELEX and SMILE to conventional LASIK [61, 65–67]. SMILE is thought to be potentially more accurate than LASIK as it is not associated with the variability of environmental factors that can influence excimer stromal ablation, such as laser fluence and differences in stromal hydration. SMILE has mainly been but not solely used in mild myopia with studies on low and high myopic corrections slowly emerging [68, 69]. Although attempts have been made to provide hyperopic SMILE treatments in a form of endokeratophakia [70, 71], these treatment modalities have yet to be standardized and made widely available.

At present, more work is needed to determine which technique is superior in cases where both techniques can be employed. LASIK has the benefit of vast surgical and research experience but newer techniques appear to be very promising not only in terms of their efficacy but also in terms of quality of life factors, such as reduced dry eye symptoms and preserved corneal sensation. There has been a tendency in recent years to document patient reported outcomes and it will be interesting to see how such factors will influence our perception of femtosecond lasers in the future [72–74].

### Femtosecond laser assisted astigmatic correction

Femtosecond lasers have been programmed and approved for the treatment of corneal astigmatism in the form of astigmatic keratotomy (AK) and intracorneal ring segments. Preliminary results in post keratoplasty patients with high corneal astigmatism have been reported, but due to scarce literature on this topic, a firm surgical protocol has yet to be developed. Nevertheless, femtosecond astigmatic correction remains an attractive possibility due to the precision provided by femtosecond lasers in placing the incisions within the corneal stroma.

#### Penetrating astigmatic keratotomy

Penetrating AK has been the mainstay of non-excimer laser assisted astigmatic correction amenable for low to high astigmatic corrections including post-keratoplastic astigmatism up to 10.0 D. The technique is performed by limbal, arcuate, or transverse incisions using diamond blades in an attempt to alter the corneal curvature. The principal advantage of this technique is the ability to correct high astigmatic errors that refractive excimer lasers cannot [75]. However, the main limitation has been the unpredictability of manual corneal incision resulting in variability in incision depth, corneal perforations, under-corrections, induced irregular astigmatism and sometimes worsening of the pre-existing astigmatism [76, 77]. The length, optical zone and depth of the incision are important parameters in predicting the magnitude of astigmatic correction. Several nomograms have been developed for example; Lindstrom’s including an adjustment for the patient’s age [78–82]. Ideally, the depth of the incision should be approximately 95 % depth or 20 μm less than the thinnest depth measured at the point of the incision. Paired astigmatic incisions, usually orthogonal across the steep axis, can be used to correct high astigmatic errors. Low astigmatic errors (≤2.75 D) can be corrected by limbal incisions (11 mm optical zone), while the addition of 8 mm optical zone incision is intended for corrections > 3.0 D. In post-keratoplasty AK, the magnitude of the corrected cylinder is mainly influenced by the magnitude of the pre-existing cylinder; therefore nomograms have less predictive capacity and corneal topography may serve as a more useful guide [83, 84].

#### Femtosecond intrastromal astigmatic keratotomy

The implementation of femtosecond laser in AK can provide enhanced precision and control over the shape, length, depth and location of the corneal incisions, and improved visual outcomes [85–88]. Thus far, femtosecond AK correction has been primarily implemented in post-keratoplastic eyes with moderate to high cylindrical refractive error (≥4.0 D) but its use in conjunction with cataract surgery is rapidly growing [86, 89]. Modification of the Donnenfeld LRI nomogram (www.lricalculator.com) at 70 % depth, Julian Stevens’s Intrastromal AK nomogram calculator (www.femtoemulsification.com), and the ASSORT Femto LRI Calculator have become popular. One major benefit of femtosecond AK incisions is that they can be penetrating- or intrastromal-only allowing titration of the astigmatic effect and less discomfort when the incisions are not opened or constructed.

Bahar et al. compared the results of manual versus femtosecond laser assisted AK in 40 eyes of 39 post-keratoplasty and found that the femtosecond AK provides significant improvement in uncorrected and best corrected visual acuity attributed to the increased accuracy and precision of femtosecond technology and the reduced complication rates [90].

The most common femtosecond AK related complications are reversible, such as self-healing micro corneal perforations and low-grade inflammation at the incision site. Thin corneas can cause overcorrection, necessitating the incorporation of corneal topography and pachymetry in the surgical planning.

In a published case report of post keratoplasty astigmatic correction using the IntraLase 30 kHz femtosecond laser, 2 anterior arcuate incisions, (60-degree arc length, from 180 to 240 degrees, and from 320 to 20 degrees) at 75 % depth of the thinnest corneal measurement were performed, which resulted in cylinder reduction from -4.0 D to -0.5 D with concomitant improvement in uncorrected and best-corrected visual acuity from 20/60 to 20/50, and 20/50 to 20/32, respectively [89]. In a case report of post keratoplasty astigmatism, correction was attempted with the IntraLase 60 kHz by placing paired arcuate cuts deep into the donor corneal button at different angles, resulting in cylinder reduction from 9.3 D to 6.5 D and uncorrected visual acuity improvement from 1.27 to 0.55 (logMAR) [91]. In a study of nine post-keratoplasty eyes of nine patients, astigmatic correction was attempted using the IntraLase 60 kHz femtosecond laser by placing two simultaneous opposite paired incisions of 70 degrees arc length at 80 % of the thinnest corneal point, centered on the steep keratometric axis with 90 degrees side cuts. The technique resulted in a significant reduction in cylinder from 9.10 (±3.90) D to 5.20 (±1.50) D (mean ± SD) and improvement in mean best corrected visual acuity from 20/30 to 20/25 [85].

Low to moderate astigmatic errors (1.0 to 3.0 D) may also be corrected by intracorneal ring segment implantation, amenable to ectatic conditions of the cornea such as keratoconus, post-LASIK ectasia and pellucid marginal degeneration. The technique aims to delay or prevent corneal grafting and it involves the manual creation of two arc shaped intracorneal tunnels or a continuous ring with the femtosecond laser at two-thirds of the corneal thickness and the implantation of plastic polymethylmethacrylate biocompatible segments peripherally in the tunnels for the mechanical flattening of the cornea. Intracorneal tunnels with femtosecond lasers are more predictable and precise thus minimizing surgical complications of manual incision, such as incomplete tunnel formation, endothelial perforation, segment extrusion or migration, corneal melting and granulomatous particles around the Intacs segments. The reported overall complication rate of the procedure was 5.7 % (49 out of 850 cases) in a study comparing femtosecond and manual intracorneal ring segment implantation [92, 93]. Both mechanical and femtosecond laser-assisted procedures provide similar visual and refractive outcomes [94].

### Femtosecond laser-assisted penetrating keratoplasty

Penetrating keratoplasty (PKP) has changed over the years, from the early 1900s, when the first procedure was performed without any modern surgical device [95] to the novel femtosecond laser-assisted PKP concept. On the other hand, optimal postoperative outcomes remain a concern as they are dependent on a centered and perpendicular cut of the recipient cornea, and a well-matched donor button and recipient bed [96]. The femtosecond laser has shown promise in improving postoperative outcomes, as it can achieve a greater precision in cutting either the donor or the recipient cornea, minimize misalignments and increase the stability of the wound [97].

The concept of a stepped graft edge dates back to 1960s, when Jose I. Barraquer described the “two-level keratoplasty” technique, characterized by a difference in the size of the graft at the level of the anterior and posterior layers of the cornea [98]. Over the years, technical complexity and manual techniques have prevented the shaped corneal grafts from becoming widely implemented [99]. However, the femtosecond laser has now enabled the creation of advanced shaped corneal cuts, eliminating manual dissection [96].

Laboratory studies in shaped corneal grafts using femtosecond lasers first demonstrated the mechanical stability of different known wound configurations, such as “top-hat”, “mushroom”, “zigzag” and “Christmas tree” [97, 100, 101]. Subsequent in vivo reports were very optimistic in describing excellent wound apposition, wound integrity and best spectacle-corrected visual acuity greater than 20/30, after 6 months, in patients who underwent PKP with femtosecond laser zigzag pattern [102]. Likewise, the first study comparing the conventional blade trephination versus the femtosecond laser generated zigzag incision, demonstrated consistently lower induced astigmatism in the laser group. The greatest difference was experienced at month 1, followed by month 3, when the average astigmatism was 4.5 D in the conventional group versus 3.0 D in the laser group (*p* = 0.018) [99].

The femtosecond laser allows for patterns and angles of incisions that are not achievable with conventional trephines [99]. A prospective study following patients for one year, showed the laser efficacy in creating precise and complex wound configurations, even when significant corneal opacity was present [103]. In addition, the typical increased wound healing area in the top-hat configuration enabled faster and secure suture removal [103].

Regular and irregular astigmatism remain a major postoperative challenge in full thickness keratoplasty. A recent study has shown favorable outcomes of “mushroom” femtosecond laser-enabled keratoplasty (M-FLEK), in contrast to conventional PKP in eyes with keratoconus. Decreased astigmatism was observed despite no significant differences in best-corrected visual acuity [104].

### Femtosecond laser-assisted penetrating keratoplasty in pediatric patients

In pediatric keratoplasty, faster visual acuity rehabilitation and easier postoperative management are crucial. Laser welding of the wound, a procedure already reported in adults undergoing cataract surgery [105], is an alternative to the conventional PKP sutures. The technique is based on a near-infrared diode laser radiation at 810 nm in combination with the topical application of indocyanine green dye to the corneal wound. The photoactivation of the agent leads to crosslinking, thereby achieving rapid wound closure with minimal side effects [105, 106]. In pediatric patients, femtosecond laser-assisted penetrating PKP keratoplasty followed by laser welding of the wound has led to suture-less surgery, with the potential to reduce the risk for suture-related endophthalmitis, decrease the need for general anesthesia for postoperative suture management, and to achieve fast visual rehabilitation after surgery [107].

### Femtosecond laser-assisted lamellar keratoplasty

The use of the femtosecond laser in deep anterior lamellar keratoplasty (DALK) allows precise identification of tissue depth and air injection, facilitating the big bubble formation [108]. Therefore, DALK with femtosecond laser offers several advantages over the manual technique, facilitating the use of scissors to cut residual stromal from Descemet membrane, in addition to allowing a more secure wound closure [108]. If Descemet membrane is perforated during the procedure, conversion to a full-thickness keratoplasty is still feasible while maintaining the benefits of the shaped corneal incision [99]. In addition, the sutures are typically removed earlier after femtosecond laser-assisted keratoplasty [99]. The disadvantage of the laser-assisted technique over the conventional technique may be due to the high cost.

Recent studies have reported the safety, efficacy and advantages of femtosecond-assisted suture less anterior lamellar keratoplasty (FALK) [109], as well as its long-term stability [110]. First described in 2008, the technique is based on precise cuts of both donor and recipient cornea, allowing a better apposition of the tissue without sutures. The absence of sutures appears to promote early visual rehabilitation with less induced astigmatism [109, 110].

New approaches to the big bubble formation have been suggested, such as the IntraBubble technique, that creates a channel in the posterior stromal, about 50 μm above the endothelium layer, by which the air injection is introduced, leading to the cleavage of the corneal tissue [111].

### Femtosecond laser-assisted endothelial keratoplasty

Early laboratory studies suggested a possible advantage of the femtosecond laser over the microkeratome in creating a less smooth surface, which could potentially improve the endothelial disc adherence to the receptor bed [112]. However, recent in vivo studies showed that microkeratome-assisted Descemet's stripping automated endothelial keratoplasty (DSAEK) led to better visual outcomes than femtosecond laser-assisted DSAEK [113]. The increased roughness at the deep intrastromal dissection surface could be associated with interface haze and unfavorable visual outcomes [113]. In addition, irregularities of the endothelial surface, most likely due to the applanation strain and corneal compression during the laser procedure, may lead to poorer visual outcomes [114]. Further studies are needed to elucidate the effectiveness of femtosecond DSAEK.

### Femtosecond laser-assisted presbyopic correction

#### Corneal inlay implantation

Current surgical therapeutics for presbyopia involves surgical intervention to the lens and cornea [115, 116]. Corneal presbyopic correction can be performed by femtosecond laser-assisted inlay implantation, a procedure that has gained attention due to the advances in flaps and pocket creation by femtosecond lasers as well as the remarkable improvements in the biomaterial technology of the inlay lenticules [117].

Presbyopic treatment with inlays is achieved by changing either the curvature of the anterior corneal surface, the refractive index of the cornea or by increasing the depth of focus without changing the anterior corneal surface [118]. The latter can be utilized using small-aperture corneal inlays, designed to increase the depth of field based on the principle of pinhole optics through the selection of central light rays and minimized refraction. This technique is the most frequently used in modern refractive surgery, as evident by the increased number of citations in the literature.

Several papers have reported the efficacy of femtosecond laser-assisted corneal inlay implantation, showing significant improvements in uncorrected near and intermediate visual acuity with minimal change in uncorrected distance visual acuity (UCDVA). This procedure provides good non-spectacle-corrected near vision for average daily activities [119, 120]. Tomita et al. reported a series of 180 eyes implanted with a small-aperture inlay (current version of the Kamra Inlay, AcuFocus, Inc., Irvine, California, USA) and treated with LASIK [121]. At six months postoperatively, both mean uncorrected near visual acuity (UCNVA) and UCDVA improved significantly. All patients had postoperative binocular UCDVA of 20/20 or better [121].

The main advantages of inlays are: the reversibility of the procedure by removal of the implant, the simplicity in implantation and repositioning of implants, and the ability to perform ad hoc refractive procedures to allow the simultaneous correction of ametropia [121–123].

The most common complaints of patients after the surgery are related to glare, halos, night vision deterioration and dry eye, which can be subjectively assessed with the Quality of Vision (QoV) questionnaire [73, 74, 121, 124]. The centration of the inlay lenticule on the visual axis is based on the Purkinje reflex and is essential to prevent postoperative symptoms and to achieve the best possible refractive results [125].

#### IntraCor treatment

IntraCor surgery is a new technique that utilizes the femtosecond laser technology to correct presbyopia by selectively changing the topographic and refractive characteristics of the central portion of the cornea [126, 127]. The technique involves the creation of several concentric intrastromal rings (5 to 8), using a femtosecond laser, at different corneal depths (between the Bowman’s and Descemet’s boundaries) in the central portion of the cornea. It is applicable to emotropic or low degree hyperopic eyes (+0.5 to +1.5 D), however, recent modifications suggest that this technique may be applicable in low myopic eye as well [128]. It is typically performed in the non-dominant and yields stable gain of UCNVA and integrity of the cornea up to 12 and 18 months postoperatively without changing the pachymetry of the cornea [128]. However, this gain may cause a loss of 1–2 lines of corrected distance visual acuity in some patients, a complication that is not always acceptable. No major surgical complications are associated with this technique other than the possibility of treatment failure in regards to presbyopic correction. This technique has already shown promising results but requires more time for maturation.

### Femtosecond laser-assisted cataract surgery

Femtosecond-laser-assisted cataract surgery (FLACS) is a new technology in the field of ophthalmology. The first implementation of femtosecond laser cataract surgery was performed in 2008 in Europe by Nagy et al. [129] and rapidly spread around the world. This technique involves the focal photodisruption of the tissue using a laser beam, typically generated at a wavelength of 1053 nm. All FLACS platforms are equipped either with an optical coherence tomography (OCT) imaging system or a Scheimpflug camera to guide the laser beam to the target. In 2010, the US Food and Drug Administration (FDA) approved this technology for capsulorhexis, lens fragmentation and liquefaction, corneal incision and arcuate corneal incisions [130].

To date, five femtosecond-laser platforms are available for cataract surgery: the LenSx (Alcon LenSx, Fort Worth, Texas), the Catalys (Optimedica Catalys, Santa Clara, CA, USA), the LensAR (LensAR Inc., Orlando, FL, USA), the Victus (Technolas Perfect Vision and Bausch and Lomb, Rochester, NY, USA) and the LDV Z8 (Ziemer Ophthalmic Systems AG, Switzerland) [131]. The key indications of femtosecond laser cataract surgery are [132]:

- Anterior capsulotomy
- Laser fragmentation of the crystalline lens (harder lenses)
- Laser liquefaction of the crystalline lens (soft lenses)
- Single plane or multiplane (uniplanar, biplanar, triplanar, etc.) corneal cuts with 2–3 incisions
- Arcuate corneal cuts to control preoperative corneal astigmatism
- Pediatric cataract (for anterior and posterior capsulotomy)

There is one relative contraindication while performing the capsulorhexis, which is the small non-dilating pupil. Ideally, the pupil size should be at least 6.0 mm in diameter. Smaller sizes increase the risk of phimosis and iris trauma. This represents a real limitation compared to manual cataract surgery where dilation can be achieved with a ring, hooks or viscoelastic in the anterior chamber. However, since the FLACS capsulorhexis is accomplished without incision, enlarging the pupil is not possible [132]. Prior to FLACS surgery, eyes with a pupil diameter smaller than 5.5 mm may receive a sequential treatment with intracameral administration of epinephrine solution, additional viscomydriasis or implantation of a Malyugin ring pupil expander. No other alterations are required and the surgery may proceed as initially planned [133, 134].

### Procedural steps

At the beginning of the procedure, the surgeon has to determine and set the optimal surgical plan. The imaging system of each platform is utilized to evaluate the anatomical characteristics of the anterior part of the eye (cornea, anterior chamber, pupil and lens). However, further parameter adjustments are necessary to define the capsulorhexis size, lens fragmentation pattern, corneal incisions and probable arcuate incision size and depth. Detailed planning of each stage of the operation is a prerequisite for a successful outcome [135].

**Fig. 2:**
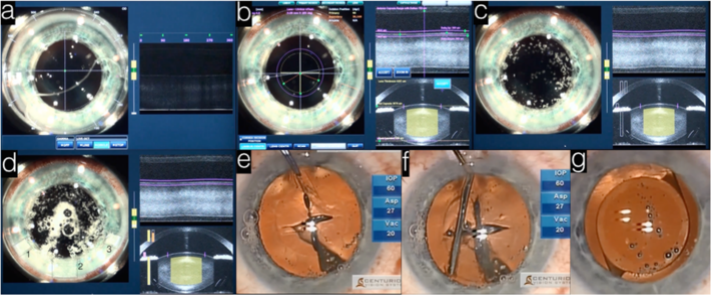
Femtosecond laser assisted cataract procedure. **a.** The eye is docked (left) using a cone and the level of the cornea is inspected (right) using live imaging. **b.** The incisions’ position is set and (**c**) the capsulorhexis phase is initiated followed by (**d**) the lens fragmentation phase. **e.** The capsule is manually removed and (**f**) the lens fragments are separated using hydrodissection. **g.** The IOL is injected and positioned in the eye, concluding the procedure

Next, the eye is docked into the laser platform in a method similar to that in excimer laser refractive surgery (Fig. 2a). Docking of the eye in FLACS causes minimal IOP elevation (~20 mmHg) as compared to LASIK docking that results in acute IOP elevation of more than 80 mmHg [136]. This advantage benefits patients with pre-existing retinal nerve fiber layer and optic nerve head pathologies, especially in cataract surgery where the mean patients’ age is higher than those patients of LASIK [137–141].

The third step in the procedure involves the acquisition of high-resolution, three-dimensional, wide-field imaging of the anterior segment (Fig. 2b). The LenSx, Catalys (Optimedica), VICTUS (Technolas Perfect Vision) and LDV Z8 (Ziemer Ophthalmic Systems AG, Switzerland) are utilizing a Fourier-domain OCT system while the LensAR uses a confocal structured illumination-scanning transmitter system that is similar to the Scheimpflug imaging developed for corneal topography [129, 142–144].

The last step involves the initiation of the laser sequence (Fig. 2c-d). Different platforms require a different sequence of procedures. For example in LenSx, the capsulorhexis is performed first, followed by the lens fragmentation and finalized by the corneal incisions (Fig. 2e-f). The lens is removed usually with modified phacoemulsification and irrigation/aspiration technique then exchanged with an artificial intraocular lens (IOL) [145] (Fig. 2g).

#### Capsulotomy

One of the most important advantages of FLACS is the ability to perform a customized capsulotomy. The size and centration of the capsulotomy is very important. Friedman et al. found increased predictability in resected capsular button diameter using laser (29 ± 26 μm deviation form intended diameter) as compared to manual capsulotomy (337 ± 258 μm) [143]. Laser capsulotomy improved IOL positioning and resulted in less IOL tilt and decentration as compared to manual continuous curvilinear capsulorhexis (CCC) [146]. In the same study, the authors noted that manifest refraction values correlated with the total IOL decentration postoperatively. Other groups have shown more accuracy in lens positioning and increased predictability in the refractive outcome using laser cataract surgery (LCS). LCS provides more stable anteroposterior and central IOL positioning with better refractive outcomes. This becomes even more important in premium IOL implantation, where patients’ expectations are high. New generation IOLs with capsule fixation will benefit from the unprecedented control over the capsulorhexis parameters provided by the laser [132, 147, 148].

#### Lens fragmentation and liquefaction

One of the limitations of the manual phacoemulsification technique is the increased delivered energy to the eye for lens fragmentation and liquefaction, which can result in energy dependent endothelial cell damage. Palanker et al. first reported a series of cases undergoing LCS with decreased nuclear hardness during phacoemulsification. The same study also reported a 39 % reduction in the cumulative dispersed energy during phacoemulsification in laser cut lenses as compared with the manual cohort [145]. A preliminary study by Nagy et al. using femtosecond laser in cataract surgery, saw a significant reduction in ultrasonic energy delivered during phacoemulsification with LCS as compared to routine surgery [129]. Whether these factors have an impact on endothelial cell loss is yet to be determined [146]. Takács et al. compared central corneal thickness and endothelial cell count between eyes undergoing LCS or conventional phacoemulsification and found that femtosecond laser-assisted cataract surgery resulted in less corneal swelling in the early postoperative period, possibly associated with reduced endothelial damage. However, no differences were found between the groups at later postoperative follow-up examinations [148].

#### Corneal incisions

Corneal incisions performed by femtosecond laser are more precise in width, depth, and length. This represents a major advantage over manual corneal surgery. Masket et al. showed in cadaver eyes that manual incisions are more deformable under pressure with increased risk for being Seidel positive after cataract surgery [149]. Arcuate or relaxing incisions are done in order to correct corneal astigmatism. They allow the cornea to change shape and correct the astigmatic error simultaneously with the lens exchange surgery. Otherwise, in cases of corneal astigmatism, a toric IOL has to be used. Literature suggests that 9 to 30 % of toric IOLs exhibit rotation by 5 or more degrees within the first 12 postoperative months. This reduces the power of the toric correction and suggests that laser-assisted corneal incisions for astigmatic correction may provide more stable and accurate long-term outcomes compared to toric IOLs [76, 150–152].

### Femtosecond in pediatric cataract surgery

During pediatric cataract surgery, the capsulorhexis step is technically more difficult to perform. The main challenge in manual capsulorhexis is the increased elasticity of the capsular bag and the unpredictability of the capsulorhexis shape. Dick et al. successful implemented FLACS to perform laser-assisted posterior capsulotomies in 4 infants aged 9 months to 7 years using the Catalys platform. The reported outcomes showed a slight enlargement in the diameter of the anterior and posterior capsulotomies, which were attributed to the increased capsular elasticity. Currently, no platforms are designed for pediatric cataract surgery; however, this is expected to be improved in the near future [153].

### Complications of femtosecond laser-assisted cataract surgery

#### Pupillary constriction

Preoperatively, the pupil should be at least 6.0 mm in diameter. During laser programming, the capsulotomy diameter should be at least 1.0 mm smaller than the pupillary diameter. Pupillary constriction may arise during the first steps of femtolaser procedure especially after docking. Bubble formation in the anterior chamber releases small amounts of free radicals that can trigger pupillary constriction [154]. Some studies have shown high levels of total prostaglandin and prostaglandin E2 in the aqueous humor during anterior capsulotomy, suggesting their contribution to pupillary constriction [155, 156]. Optimizing the energy setting and administering a non-steroidal anti-inflammatory therapy may help to circumvent this reaction and laser-induced miosis [155, 156]. Surgeons should also minimize the delay between femtolaser pretreatment and cataract surgery as this may result in pupil diameter changes [132, 154].

#### Capsular blockage syndrome

Introducing high-speed fluid with a large diameter hydrodissection cannula may inhibit the gas bubble that is formed from leaving the nucleus. Rupture of the posterior capsule may be caused by pressure elevation between the capsule and lens causing the nucleus to drop into the vitreous cavity. Surgeons should be aware of the capsular blockage syndrome complication and perform precise and careful maneuvers during the lens-dissection step in order to avoid it [154–158].

#### Corneal incision sizing and positioning

The initial docking of the laser ring is very important for the accuracy of the intended incisions, capsulorhexis, and lens fragmentation. During the first set up step of the planning procedure, the incisions are set to the correct position automatically. Moreover, the user can set up the incisions manually. If they are set centrally to the cornea, this may cause corneal astigmatism, and if set peripherally to the cornea, may complicate the manual final procedure. Regardless of the position of the incisions, dilation with a spatula before entering the eye is generally advised [154, 159–161].

## Conclusion

Femtosecond laser technology has revolutionized everyday ophthalmic practice. The implementation of this technology in corneal refractive surgery has dramatically improved the safety, efficacy, and predictability of LASIK flaps. Further advancements were also achieved when femtosecond technology was introduced to non-refractive corneal surgery, such as anterior and posterior lamellar keratoplasty, to perform high precision cuts in the donor and host corneal tissue. Femtosecond technology is still under assessment for non-LASIK corneal refractive procedures e.g. SMILE, and for the correction of astigmatism and presbyopia, with initial reported results being highly encouraging. Important advancements were also achieved in the field of cataract surgery using femtosecond technology especially in anterior capsulotomy, lens fragmentation, and corneal incision. Some major limitations still exist, such as the small, non-dilating pupil, however, femtosecond lasers are very promising. As with any new technology, the execution of the surgical procedure requires optimization and customization. Nevertheless, the technological advancements over the past few years have brought significant software and hardware improvements resulting in greater surgical flexibility and precision.
